# Voluntary Hospitals

**Published:** 1921-02-12

**Authors:** 


### Voluntary Hospitals.

### THE CASE FOR PAYING PATIENTS.

A correspondent, "C.L.," says he has read with gratitude a recent article in these columns which stated the case of the poor mididle-class patient, and suggested that the Committee appointed to inquire into the general position of the voluntary hospitals should investigate with special care and sympathy the full possibilities of a system of paying patients in order to assist the hard lot of the middle classes as well as to meet the financial problem of the hospitals. This correspondent asserts that there is nothing which strikes the imagination of the professional or business family man of moderate means with more terror than the fear of serious sickness, because he is able to allow no margin whatever for doctor's fees. "I am a man with £500 a year," he writes, "and a wife and three small children. We live in a small house, on the most economical basis, and our only adult indulgences are my weekly two ounces of tobacco and an occasional box of chocolates for my wife. We have not even taken a holiday away from home since the war ended; yet I am now insolvent entirely through sickness. We have had nothing but illness in the house during the past twelve months, beginning with a dangerous attack of influenza which nearly killed my wife and has partly incapacitated her ever since; and doctor's bills and the other inevitable extras in illness have swallowed up all my small bank balance and caused me to get into debt with tradesmen. I wish now that I had followed my first instinct and allowed my wife to go into hospital. Yet I realise that it would

**THE PUBLIC WELL-BEING—(*continued*).**

be taking an unfair advantage of a charitable institution, though I should not hesitate if a system were established which compelled every patient to pay according to his means."

"C.L." goes on to suggest that if such a system is introduced it should be based on the amount of income-tax paid by the applicant for admission. We do not think this is an altogether satisfactory proposal. Indeed it possesses many serious disadvantages, not the least of which is that it is by no means a safe guide to a man's actual financial position. It should not be difficult to arrange a fair and properly graduated method of payment. But the point which it is important to bear in mind is that there is evidently a strong body of opinion favourable to the paying-patient principle, and if the Committee appointed by the Government, when all the cases are before it, regards sympathetically the continuance of the voluntary idea, we do not see how the application of this principle can be left out of account.

There is some reason to believe that the Ministry of Health would not be at all averse from leaving the voluntary system intact if some *modus vivendi* could be found. Sir Kingsley Wood, in a public speech, spoke the other day quite encouragingly of the value of a paying-patient system, and he also made some useful observations upon the possibilities of a more scientific and comprehensive method of collecting regular contributions to the upkeep of the hospitals. It is the writer's opinion, based on the success of a contribution scheme now established in respect of a northern hospital, that by means of a definite organised scheme of appeal sufficient public funds could be raised annually, with steadily decreasing difficulty, to maintain every hospital in the country as a voluntary institution, provided always that patients pay for admission according to their means, that a more rigid control is exercised over administrative expenditure, and that the proposals already formulated for correlating the work of hospitals and preventing overlapping are carried out in a practical form.

